# Respiratory Drive, Effort, and Lung-Distending Pressure during Transitioning from Controlled to Spontaneous Assisted Ventilation in Patients with ARDS: A Multicenter Prospective Cohort Study

**DOI:** 10.3390/jcm13175227

**Published:** 2024-09-03

**Authors:** Eleonora Balzani, Francesco Murgolo, Matteo Pozzi, Rossella Di Mussi, Nicola Bartolomeo, Umberto Simonetti, Luca Brazzi, Savino Spadaro, Giacomo Bellani, Salvatore Grasso, Vito Fanelli

**Affiliations:** 1 Department of Surgical Sciences, University of Turin, 10126 Turin, Italy; eleonora.balzani@unito.it (E.B.); luca.brazzi@unito.it (L.B.); 2 Department of Precision-Regenerative Medicine and Jonic Area (DiMePRe-J), Section of Anesthesiology and Intensive Care Medicine, University of Bari “Aldo Moro”, 70010 Bari, Italy; francesco.murgolo@uniba.it (F.M.); rosydimussi87@gmail.com (R.D.M.); salvatore.grasso@uniba.it (S.G.); 3 Department of Emergency and Intensive Care, IRCCS San Gerardo dei Tintori Foundation, 20900 Monza, Italy; mateo.pozzi@gmail.com; 4 Interdisciplinary Department of Medicine, University of Bari Aldo Moro, 70121 Bari, Italy; nicola.bartolomeo@uniba.it; 5 Department of Anesthesia, Critical Care and Emergency, Città della Salute e della Scienza Hospital, University of Turin, 10126 Turin, Italy; umbesim@gmail.com; 6 Department of Translational Medicine, University of Ferrara, 44121 Ferrara, Italy; savinospadaro@gmail.com; 7 Azienda Ospedaliera-Universitaria di Ferrara, 44122 Ferrara, Italy; 8 Centre for Medical Sciences—CISMed, University of Trento, 38122 Trento, Italy; giacomo.bellani@apss.tn.it; 9 Department of Anesthesia and Intensive Care, Santa Chiara Hospital, 38122 Trento, Italy

**Keywords:** respiratory drive, inspiratory effort, lung-distending pressure acute respiratory distress syndrome, respiratory monitoring

## Abstract

**Objectives**: To investigate the impact of patient characteristics and treatment factors on excessive respiratory drive, effort, and lung-distending pressure during transitioning from controlled to spontaneous assisted ventilation in patients with acute respiratory distress syndrome (ARDS). **Methods**: Multicenter cohort observational study of patients with ARDS at four academic intensive care units. Respiratory drive (P_0.1_), diaphragm electrical activity (EAdi), inspiratory effort derived from EAdi (∆Pmus_EAdi_) and from occlusion of airway pressure (∆Pocc) (Pmus_ΔPocc_), and dynamic transpulmonary driving pressure (ΔP_L,dyn_) were measured at the first transition to assisted spontaneous breathing. **Results**: A total of 4171 breaths were analyzed in 48 patients. P_0.1_ was >3.5 cmH_2_O in 10%, EAdi_PEAK_ > 15 µV in 29%, ∆Pmus_EAdi_ > 15 cmH_2_O in 28%, and ΔP_L,dyn_ > 15 cmH_2_O in 60% of the studied breaths. COVID-19 etiology of ARDS was the strongest independent risk factor for a higher proportion of breaths with excessive respiratory drive (RR 3.00 [2.43–3.71], *p* < 0.0001), inspiratory effort (RR 1.84 [1.58–2.15], *p* < 0.0001), and transpulmonary driving pressure (RR 1.48 [1.36–1.62], *p* < 0.0001). The P/F ratio at ICU admission, days of deep sedation, and dose of steroids were additional risk factors for vigorous inspiratory effort. Age and dose of steroids were risk factors for high transpulmonary driving pressure. Days of deep sedation (aHR 1.15 [1.07–1.24], *p* = 0.0002) and COVID-19 diagnosis (aHR 6.96 [1–48.5], *p* = 0.05) of ARDS were independently associated with composite outcome of transitioning from light to deep sedation (RASS from 0/−3 to −4/−5) or return to controlled ventilation within 48 h of spontaneous assisted breathing. **Conclusions**: This study identified that specific patient characteristics, including age, COVID-19-related ARDS, and P/F ratio, along with treatment factors such as the duration of deep sedation and the dosage of steroids, are independently associated with an increased likelihood of assisted breaths reaching potentially harmful thresholds of drive, effort, and lung-distending pressure during the initial transition to spontaneous assisted breathing. It is noteworthy that patients who were subjected to prolonged deep sedation under controlled mechanical ventilation, as well as those with COVID-19, were more susceptible to failing the transition from controlled to assisted breathing.

## 1. Introduction

Vigorous inspiratory efforts are associated with elevated global transpulmonary pressure [1] and local stress in the solid-like-behavior atelectatic regions [2]. This heightened mechanical stress increases the risk of lung injury [3] especially in ARDS patients with low compliance and high dead space fraction, as seen in those with severe COVID-19-associated pneumonia [4,5,6]. Furthermore, excessive inspiratory effort [7] can result in load-induced diaphragm injury and weakness [8], thereby increasing the risk of dependence on mechanical ventilation and a longer ICU stay [9]. Monitoring respiratory drive, inspiratory effort, and lung-distending pressure in patients with de novo acute respiratory failure under assisted breathing may facilitate the identification of optimal ventilation settings, thereby reducing the risk of patient-induced lung injury and myo-diaphragmatic trauma [10,11,12]. Airway occlusion pressure (P_0.1_) estimates respiratory drive during mechanical ventilation. Values exceeding 3.5 have been correlated with excessive respiratory effort [13,14]. The electrical activity of the diaphragm (EAdi) can be measured via an oesophageal catheter, which allows for the estimation of the neuromechanical efficiency (NME or PEI index). This is defined as the amount of pressure that the respiratory muscles (∆Pmus) are able to generate for each µV of electrical activity [15]. The PEI value obtained during a hold end-expiratory maneuver allows for the estimation of ∆Pmus from EAdi (∆Pmus_EAdi_) [15]. Recently, the calculation of ∆Pmus (∆Pmus_∆Pocc_) and dynamic transpulmonary driving pressure (ΔP_L,dyn_) from the airway pressure swing during a hold expiratory and inspiratory maneuver has also been described [16]. The duration of controlled mechanical ventilation [17], the intensity of sedation [18], and the severity of lung injury have been demonstrated to have a negative impact on the inspiratory flow and pressure-generating capacity of patients [13]. It has been demonstrated that different stimuli, which are not always dependent on ventilatory assistance, may impact the inspiratory drive and effort of patients with ARDS [11]. These stimuli include lung inflammation and impairment of respiratory mechanics, even in the absence of impaired gas exchange [11]. A P_0.1_ value higher than 3.5 cmH_2_O, in conjunction with an excessive respiratory effort, has been associated to the recurrence of hypoxemic respiratory failure in critically ill patients with COVID-19-associated ARDS (CARDS), [19,20]. It is possible to provide protective ventilation to the diaphragm by adjusting the level of inspiratory support in line with the patient’s effort, without affecting the tidal volume or transpulmonary pressures [21]. Nevertheless, the respiratory drive and inspiratory effort of critically ill patients undergoing a transition from controlled to assisted ventilation remain poorly understood. The present study aims to investigate the magnitude of respiratory drive, effort, and lung-distending pressure in patients with ARDS who are transitioning from controlled to assisted spontaneous mechanical ventilation. We evaluated the association of patient characteristics and treatment factors with the proportion of assisted breaths that exceeded pre-defined injurious thresholds for inspiratory drive (P_0.1_ > 3.5 cmH_2_O), effort (∆Pmus_EAdi_ > 15 cmH_2_O), and transpulmonary driving pressure (ΔP_L,dyn_ > 15 cmH_2_O). To ascertain whether patients’ baseline characteristics and treatment factors influence the success of spontaneous breathing over time, a composite outcome was estimated, comprising the rate of transition from light to deep sedation (Richmond Agitation-Sedation Scale, RASS from 0/−3 to −4/−5) or the resumption of controlled ventilation within 48 h of spontaneous assisted breathing.

## 2. Materials and Methods

### 2.1. Study Population and Setting

This multicenter study was carried out in four tertiary academic intensive care units (ICUs) in Italy from March 2020 to January 2022. The institutional review board of each participating hospital approved the study and waived the requirement for informed consent. Patients were eligible for enrollment if they had a diagnosis of acute respiratory distress syndrome, also due to confirmed COVID-19 (real-time RT-PCR on nasopharyngeal swabs, or lower respiratory tract aspirates) [15,22,23], had received invasive mechanical ventilation for more than 72 h, and were candidates for assisted ventilation. The criteria for defining the readiness for assisted ventilation were as follows [24]: (a) improvement in the condition leading to acute respiratory failure; and (b) positive end-expiratory pressure (PEEP) lower than 10 cmH_2_O and inspiratory oxygen fraction (FiO_2_) lower than 0.5. Furthermore, the Richmond Agitation-Sedation Scale (RASS) score must be between 0 and −2, and the patient must be able to trigger the ventilator, that is to say, to decrease the pressure on the airway opening (PAO) by >3–4 cmH_2_O during a brief (5–10 s) end-expiratory occlusion test. Additionally, the patient was required to demonstrate hemodynamic stability without the administration of vasopressors or inotropes, with the exception of a dobutamine and norepinephrine infusion at a rate of less than 5 mcg/kg/min and 0.3 mcg/kg/min, respectively. Normothermia was also a prerequisite. Patients were excluded from the study if they exhibited neurological or neuromuscular pathology and/or known phrenic nerve dysfunction, or if they presented with any contraindication to the insertion of a nasogastric tube, such as recent upper gastrointestinal surgery or oesophageal varices.

### 2.2. Measurements and Study Protocol

Patients were ventilated using a Servo-I or a Servo-U ventilator (Maquet Critical Care, Solna, Sweden), equipped with NAVA^®^ module and a 16 Fr, 125 cm, nasogastric EAdi catheter (Maquet Critical Care, Solna, Sweden) was placed. Airway pressure (Paw), airflow, and EAdi signals were recorded for a 10 min study period at the first transition from control to pressure support ventilation at a level of sedation corresponding to a RASS scale of 0/−2. Two end-expiratory airway occlusions were applied for the duration of a single breath. Signals were acquired at 100 Hz from the ventilator to a computer using commercially available software (NAVA Tracker software 4.2, Maquet Critical Care, Solna, Sweden) and subsequently converted and analyzed using the ICU Lab software 2.9 package (Kleistek Engineering, Bari, Italy). The peak airway opening pressure (PawPEAK) and positive end-expiratory pressure (PEEP) were determined from the Paw signal. Tidal volume (VT) was calculated as the area under the inspiratory flow curve. The mechanical respiratory rate (RRMECH) was determined by analysis of the flow and Paw signals. The mechanical inspiratory and expiratory time (Ti,MECH and Te,MECH, respectively) were determined from the flow signal. Similarly, the peak EAdi (EAdiPEAK), neural inspiratory time (Ti,NEUR), and neuroventilatory efficiency (NVE), defined as the diaphragmatic ability to convert EAdi into inspired volume (VT/EAdiPEAK), were determined from the EAdi signal [24]. Respiratory drive was defined as P_0.1_, which is the decrease in Paw at 100 ms during an end-expiratory occlusion [25]. Respiratory effort was calculated as Pmus_Eadi_, which is the product of diaphragmatic electrical activity (EAdi) and PEI_occl_ (also referred to as neuromechanical efficiency—NME) [15], the ratio between the peak negative value in airway pressure of a single inspiratory effort (recorded during a 2–3 s end-expiratory occlusion) and the corresponding EAdiPEAK is defined as PEIoccl. The inspiratory pressure–time product of the Pmus_EAdi_ per breath (Pmus_EAdi_/b), which is a measurement of the work of breathing, was calculated as the area under the Pmus_EAdi_ signal. The Pmus_EAdi_ per minute (Pmus_EAdi_/min) was calculated as the product of Pmus_EAdi_/b and respiratory rate (RR). Respiratory effort was also calculated as Pmus_∆Pocc_, which is the change in Paw from PEEP during an end-expiratory occlusion (ΔPocc) multiplied by 0.75 [16]. In addition, the dynamic transpulmonary driving pressure (ΔP_L,dyn_), which is a measure of lung stress, was calculated as follows: (Paw_PEAK_ − PEEP) − 0.66 × ∆Pocc [16]. The baseline characteristics of the patients included age, sex, the presence of a coexisting chronic disease, weight, and height, which were used to calculate the body mass index (BMI) and the predicted body weight (PBW). The initial severity of the patients was assessed using the SAPS II score, and organ failure was assessed using the SOFA score. Prior to the commencement of the study, a comprehensive range of respiratory parameters were recorded, including tidal volume, positive end-expiratory pressure, plateau pressure, driving pressure, and the PaO_2_/FiO_2_ ratio. Additionally, arterial blood gas analysis was conducted, along with an assessment of the utilization of rescue therapies, such as prone positioning, inhaled nitric oxide (iNO), lung recruitment maneuvers (LRM), the duration of mechanical ventilation, the administration of sedatives, muscular paralysis, and the dosage of steroids. Patients were monitored until either hospital discharge or death. The rate of transition from light to deep sedation (RASS from 0/−3 to −4/−5) or from assisted to controlled ventilation was evaluated within 48 h of assisted spontaneous breathing. Patients were transitioned from assisted to controlled mechanical ventilation or deeper sedation at the discretion of the attending physician, typically in the presence of one or more of the following conditions: a PSV level > 20 cmH_2_O or a PSV level PEEP > 30 cmH_2_O, dyspnoea, diaphoresis, or paradoxical breathing. The following parameters were monitored: breathing, use of accessory respiratory muscles, necessity for neuromuscular blockade and/or deep sedation, hypoxemia (defined as a PaO_2_ ≤ 60 mmHg or SpO_2_ ≤ 90% or requirement for FiO_2_ ≥ 0.60), or respiratory hypercapnia with pH lower than 7.35, and hemodynamic instability [23].

### 2.3. Statistical Methods

Continuous variables were reported as mean (SD) for normal distributed parameters or as median (interquartile range [IQR]) otherwise, and Shapiro–Wilk and Kolmogorov–Smirnov tests were used to check normality. Categorial parameters were reported as absolute and relative frequencies. The goal of the analysis was to analyze the respiratory drive, inspiratory effort, and transpulmonary driving pressure of a convenient sample size of patients equipped with nasogastric EAdi catheter. We applied the Generalized Estimated Equation (GEE) model to estimate the average number of breaths in each of three pre-defined classes of respiratory drive (“Low”: P_0.1_ < 1 cmH_2_O, “Normal”: P_0.1_ 1–3.5 cmH_2_O, and “High”: P_0.1_ > 3.5 cmH_2_O), neuroventilatory drive (“Low”: EAdi_PEAK_ < 5 µV, “Normal”: EAdi_PEAK_ 5–15 µV, and “High”: EAdi_PEAK_ > 15 µV), muscle effort (“Normal”: ∆Pmus_EAdi_ < 15 cmH_2_O and “High”: ∆Pmus_EAdi_ > 15 cmH_2_O), diaphragm efficiency (“Low”: PTP/min < 50 cmH_2_O/s/min, “Normal”: PTP/min 50–150 cmH_2_O/s/min, and “High”: PTP/min > 150 cmH_2_O/s/min) and transpulmonary driving pressure (“Low”: ΔP_L,dyn_ < 15 cmH_2_O and High: ΔP_L,dyn_ > 15 cmH_2_O) [12]. In the GEE model, a single breath is the first level unit, the number of breaths is the dependent variable, the class of P_0.1_, EAdi, ∆Pmus_EAdi_, PTP/min is the independent variable and, finally, the patient is the second-level unit. Pairwise comparisons between the estimated time spent in each class were adjusted according to Tukey. We evaluated the effect of patients’ baseline characteristics (age, sex, COVID-19 diagnosis for ARDS, patient severity SOFA, P/F ratio, respiratory system compliance) and treatment factors (days of sedation, days of NMBA, dose of steroids, days of mechanical ventilation) before transition to assisted ventilation on the proportion of breaths at higher class of respiratory drive (P_0.1_ > 3.5 cmH_2_O), inspiratory effort (∆Pmus_EAdi_ > 15 cmH_2_O), and transpulmonary driving pressure (ΔP_L,dyn_ > 15 cmH_2_O), by a multivariable Poisson regression model. Parameters with *p*-values lower than 0.15 at univariate analysis were initially included in the multivariate model and a backward selection method based on *p*-value criterion (threshold *p* < 0.15) was used to estimate the final model. The results were expressed as rate ratio (RR) and 95% confidence interval.

Additionally, we applied a univariate and multivariable Cox-regression model to estimate the association of patients’ baseline characteristics (age, sex, COVID-19 risk factor of ARDS, patient severity SOFA, P/F ratio, respiratory system compliance) and treatment factors (days of sedation, days of NMBA, dose of steroids, days of mechanical ventilation) before transition to assisted ventilation with a composite outcome of transitioning from light to deep sedation (RASS from 0/−3 to −4/−5) or from assisted to controlled ventilation within 48 h of spontaneous assisted breathing. The proportional hazard assumptions for the Cox model were checked and results were expressed as hazard ratios (HRs) and their 95% confidence interval. A *p*-value < 0.05 was considered statistically significant. Analyses were performed using SAS/STAT^®^ Statistics version 9.4 (SAS Institute, Cary, NC, USA) and Rstudio, Poist Software, PBC, Version 2023.

## 3. Results

### 3.1. Patients Baseline Characteristics

Forty-eight patients were enrolled in the study over sixty-seven eligible, at four participating centers (Figures S1 and S2). Patients’ characteristics at baseline are shown in Table 1. Seventy percent of patients were male and COVID-19 pneumonia was the risk factor of ARDS in thirty-three percent. Rescue therapies were applied in more than half of patients (Table S1). The median duration of deep sedation and muscular paralysis was four days. Steroids were given to 79% of patients (Table S1). The transition from controlled to spontaneous assisted ventilation was ten days after ICU entry. The proportion of patients who went from assisted to controlled ventilation within 48 h was 6.3% or needed higher sedation was 18.8%. The mortality rates in ICU and at 60 days were 8.3 and 6.3%, respectively (Table 1).

### 3.2. Ventilator Settings, Respiratory Drive, and Effort during Spontaneous Assisted Breathing

**Figure 1:**
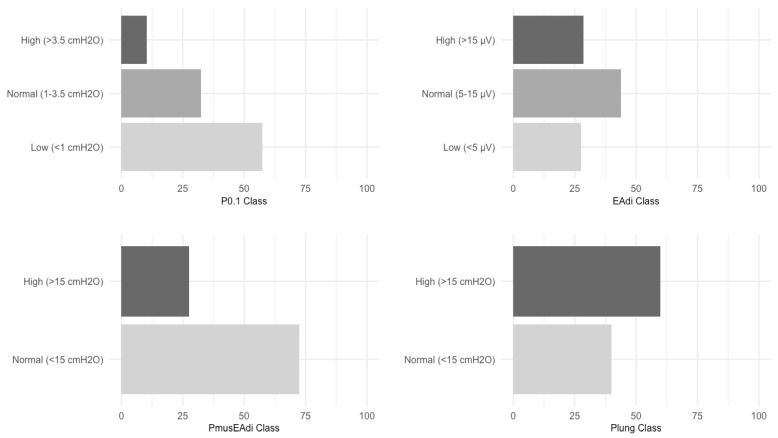
Percentage of breaths in “Low” (P_0.1_ < 1 cmH_2_O), “Normal” (P_0.1_ 1–3.5 cmH_2_O), and “High” (P_0.1_ > 3.5 cmH_2_O) respiratory drive classes (**top left**). Percentage of breaths in “Low” (EAdi_PEAK_ < 5 µV), “Normal” (EAdi_PEAK_ 5–15 µV), and “High” (EAdi_PEAK_ > 15 µV) neuroventilatory drive classes (**top right**). Percentage of breaths in the “Normal” (∆Pmus_EAdi_ < 15 cmH_2_O) and “High” (∆Pmus_EAdi_ > 15 cmH_2_O) classes of respiratory effort (**bottom left**). Percentage of breaths in the “Normal” ΔP_L,dyn_ < 15 cmH_2_O) and “High” ΔP_L,dyn_ > 15 cmH_2_O) classes (**bottom right**).

A total of 4171 breaths from the 48 patients were analyzed throughout the study period (Figure S1). A total of 10 and 29% of the examined breaths had values of P_0.1_ (median value of 0.8 cmH_2_O) and EAdi peak (median value of 9.6 µV) (Table 2) in the highest class of respiratory drive (P_0.1_ > 3.5 cmH_2_O and EAdi > 15 µV) (Figure 1 and Figure S3). Inspiratory effort expressed as ∆Pmus_ΔPocc_ (median value of 7.9 cmH_2_O) and PTP EAdi/min (median value of 95.6 cmH_2_O/s/min) (Table 2) was in the higher class (∆Pmus_EAdi_ > 15 cmH_2_O and PTP EAdi/min > 150 cmH_2_O/s/min) in 28% and 20% of the examined breaths (Figure 1 and Figure S4). Dynamic transpulmonary driving pressure (ΔP_L,dyn_) (median value of 16.2 cmH_2_O) (Table 2) was in the higher class (ΔP_L,dyn_ > 15 cmH_2_O) in 60% of the examined breaths (Figure 1).

### 3.3. Effects of Patients’ Baseline Characteristics and Treatment Factors before Transition to Assisted Ventilation on Respiratory Drive, Inspiratory Effort, and Lung-Distending Pressure

**Figure 2:**
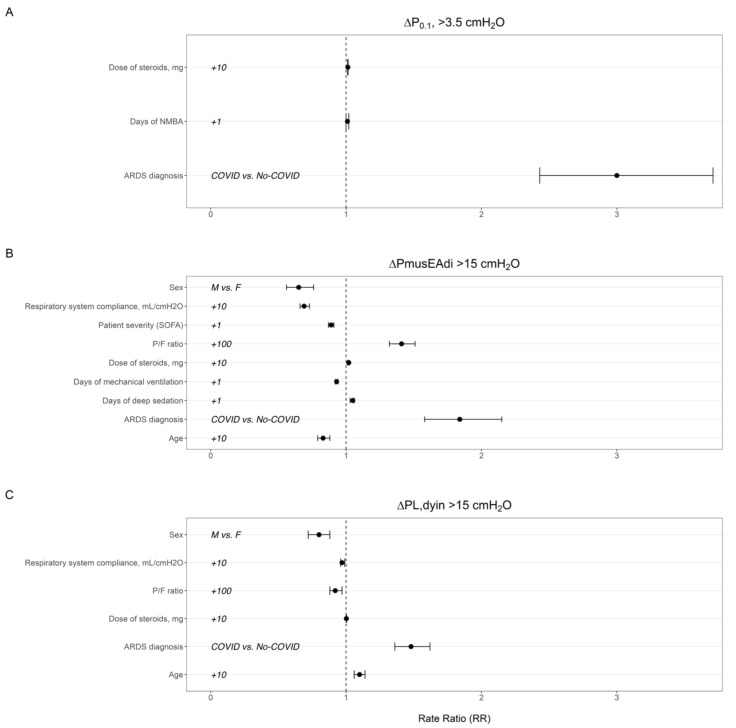
Rate ratio of treatment factors and patients’ baseline characteristics accounting for proportion of breaths in a higher class of respiratory drive (P_0.1_) (panel **A**), inspiratory effort (∆Pmus_EAdi_) (panel **B**), and lung-distending pressure (ΔP_L,dyn_) (panel **C**).

COVID-19-associated ARDS and the dose of steroids were independent risk factors of the proportion of breaths in the higher class of respiratory drive (P_0.1_ > 3.5 cmH_2_O) (RR 3 [CI 95%, 2.43–3.71] and 1.01 [CI 95%, 1.011–1.015], respectively; *p* < 0.0001) (Figure 2A). P/F ratio at ICU admission, COVID-19-associated ARDS, days spent under deep sedation, and the dose of steroids were associated with a higher proportion of breaths in the higher class of inspiratory effort (∆Pmus_EAdi_ > 15 cmH_2_O) (RR 1.41 [CI 95%, 1.32–1.51], 1.84 [CI 95%, 1.58–2.15], 1.05 [CI 95%, 1.03–1.06], and 1.02 [CI 95%, 1.01–1.02], respectively, *p* < 0.0001) (Figure 2B and Table S2). Male sex, age, respiratory system compliance, and SOFA score were all variables associated with a lower proportion of breaths at a high intensity of effort (Figure 2B and Table S2). Age, COVID-19 risk factor of ARDS and the dose of steroids were associated to higher proportion of breaths at a high transpulmonary driving pressure (ΔP_L,dyn_ > 15 cmH_2_O) (RR 1.10 [CI 95%, 1.06–1.14], 1.48 [CI 95%, 1.36–1.62], and 1.002 [CI 95%, 1.001–1.004], respectively, *p* < 0.05) (Figure 2C and Table S2). Male sex, P/F ratio, and respiratory system compliance were all variables associated with a lower proportion of breaths at a high transpulmonary driving pressure (Figure 2C and Table S2).

### 3.4. Effects of Patients’ Baseline Characteristics and Treatment Factors on Need for Sedation and Resuming Control Ventilation

According to a multivariable Cox regression model, days of deep sedation (aHR 1.15 [1.07–1.24], *p* = 0.0002) before transition to assisted spontaneous breathing and COVID-19 diagnosis of ARDS (aHR 6.96 [1–48.5], *p* = 0.05) were independent risk factors for the composite outcome of transitioning from light to deep sedation (RASS from 0/−3 to −4/−5) or from assisted to controlled ventilation within 48 h of spontaneous assisted breathing (Table 3).

## 4. Discussion

The major findings of this study are that patient characteristics (age, COVID-19 etiology of ARDS, P/F ratio) and treatment factors (duration of deep sedation and dose of steroids) were independently associated with a higher proportion of assisted breaths at potentially injurious thresholds of drive, effort, and lung-distending pressure in patients with ARDS during the first transition to spontaneous assisted breathing. Patients who had spent more days on deep sedation under controlled mechanical ventilation and those with COVID-19 were more likely to fail the transition from controlled to assisted breathing. Our study underscores the vital importance of meticulous monitoring and management of inspiratory efforts in ARDS patients. Balancing the advantages of spontaneous breathing with the potential risks of lung and diaphragm injury necessitates a nuanced approach that may entail modifying respiratory support and sedation strategies, tailored to the specific characteristics of patients. Despite the similarities in respiratory system compliance, plateau pressure, driving pressure, and mortality rate observed between patients with COVID-19-associated ARDS and previously published cohorts of ARDS patients [4,5,6], our findings indicate that the underlying etiology of the disease is associated with potentially injurious spontaneous breathing and early failure of transition from controlled to spontaneous breathing. P_0.1_ has been demonstrated to be a reliable bedside tool to monitor respiratory drive [25,26]. A value higher than 3.5 cmH_2_O has been associated with potentially injurious respiratory effort [25] and with relapse of respiratory failure in patients with COVID-19 [19,20]. In patients with ARDS, inspiratory drive and effort are influenced by several inputs that are not necessarily dependent on ventilatory support [11]. Even in the presence of normal gas exchange, respiratory drive may be affected by varying degrees of lung inflammation and impairment of respiratory mechanics. Specifically, COVID-19 infection involves the central nervous system as demonstrated by the presence of SARS-CoV-2 RNA and protein in the primary respiratory and cardiovascular control center in the medulla oblongata [27]. This could explain our data in patients with CARDS, who had a higher proportion of breaths at high thresholds of P0.1 and EAdiPEAK compared to No-COVID-19 patients, despite similar blood gas exchange and respiratory system elastance. COVID-19 diagnosis of ARDS was also a risk factor for breaths at excessive effort and lung-distending pressure that may play a significant role in worsening lung injury especially in the presence of low lung compliance and high dead space fraction [4,5].

EAdi allows quantification of the neural respiratory drive [15,23]. “Low” and/or “High” neuroventilatory drive have been shown in critically ill patients, irrespective of the level of assistance during spontaneous breathing [23]. In our cohort, the median value of EAdi was lower than the 10 µV that is considered a normal value for healthy subjects, however, more than 25% of breaths were at a high threshold of intensity (>15 µV). High EAdi values may be due to diaphragm weakness, overcoming respiratory load, or increased metabolic demand [28]. The relation between EAdi amplitude and breathing effort is not linear but depends on the neuroventilatory coupling [13]. However, EAdi allows computation of neuroventilatory efficiency (NVE), which is the ability to generate volume normalized to neural drive during tidal breath (Vt/EAdi). Low NVE has been associated with an unsuccessful spontaneous breathing trial and extubation failure in a heterogeneous cohort of critically ill patients, including COPD and lung transplant recipients [28,29,30,31]. In some patients, we observed a low neuroventilatory efficiency (NVE) despite a high electrical activity of the diaphragm, suggesting that these patients performed strenuous inspiratory efforts. We adopted two new methods that have been recently validated to continuously assess patients’ inspiratory effort: (1) the neuromechanical efficiency (NME) [28], also known as PEI (∆Pmus/EAdi index), which is the ratio between the amount of pressure that respiratory muscle of the patients are applying for each µV of electrical activity [15,28]; and (2) muscle pressure derived from the value of airway pressure deflection during an instantaneous hold expiratory occlusion maneuver (ΔPocc) [16], which allows also the measurement of dynamic transpulmonary driving pressure (ΔP_L,dyn_) [16]. In our study, respiratory muscle pressures derived by both EAdi traces and ΔPocc were higher in 28% and 15% of study breaths, respectively. The values of the integral of the electrical activity of the diaphragm (pressure–time products per minute—PTP/min), which is the energy expenditure of the diaphragm, were higher than 150 cmH2O/s/min in more than 20% of study breaths. In our study, steroid dosage was associated with increased breathing effort. Given the known myopathic effects of steroids, this unexpected finding can be explained by the fact that receiving higher dose of steroids is a proxy of COVID-19 patients who received higher doses of steroids and had more effortful breathing at elevated levels of transpulmonary pressure. An alternative hypothesis to consider is that steroids may reduce the ventilation-induced diaphragmatic dysfunction by exerting their anti-inflammatory effect [32]. Conversely, days of mechanical ventilation were associated with a lower proportion of breaths exhibiting excessive effort, which may be indicative of muscle weakness. Notably, the duration of deep sedation emerged as an additional risk factor for strenuous inspiratory effort. By blunting drive and effort, sedation minimizes the risk of self-induced lung injury and myotrauma [12], but exposes patients to prolonged weaning from mechanical ventilation [33] and death in ICU [34]. Indeed, sedation has the greatest impact on diaphragm activity and on the chances of being liberated from mechanical ventilation [33]. Doses of sedative infusion in the first 24 h of mechanical ventilation are associated with delayed resumption of EAdi activity, which takes a median time of up to 22 h to be resumed [35]. Our data suggest that the sedation scales used to assess the level of sedation in patients may lack specificity in the presence of dynamic patient response and inter-rater variability [36] since the duration of deep sedation (at RASS ≤ −3) was associated with high failure of transition from control to assisted breathing likely in presence of excessive efforts.

Lung-distending pressures were at potentially injurious thresholds in 60% of breaths, exposing patients to the risk of excessive lung stress and strain and load-induced diaphragm injury. A recent trial showed that “diaphragm-protective” ventilation, which consisted of a transdiaphragmatic pressure of between 3 and 12 cmH2O, was feasible without affecting tidal volumes, transpulmonary pressures, and biomarkers for lung injury [21]. Age was associated with a significant proportion of breaths at injurious lung-distending pressures. Age is a significant risk factor for mortality and susceptibility to ventilator-induced lung injury (VILI) as older adults have decreased lung compliance, which can lead to higher transpulmonary pressures [37]. Respiratory system compliance is the same in males and females, but females have higher recoil pressure, which depends on differences in lung size and maximum distending forces [38]. In the current study, male patients had a lower proportion of breaths at a high threshold of lung-distending pressure, highlighting the risk that females are more susceptible to VILI.

The current study has several limitations; firstly, effort was derived by EAdi and ∆Pocc, whereas the pressure–time product of the respiratory muscles was not measured by oesophageal pressure [39]. During the resolution phase under assisted mechanical ventilation, the esophageal catheter is the gold standard for measuring inspiratory effort and transpulmonary driving pressure (the difference between airway pressure and esophageal pressure). In a recent study, Tonelli and colleagues demonstrated that the magnitude of inspiratory effort relief, as assessed by the swing in esophageal pressure within the initial two hours of non-invasive ventilation (NIV), was an early and accurate predictor of NIV failure at 24 h [7]. However, the insertion of an esophageal catheter is a time-consuming process that requires dedicated equipment. However, all methods used in the current study have been fully validated in previous studies [15,16,25]. Secondly, the sample size was small because it was limited to patients equipped with an EAdi catheter; thus, inferences from this study are limited. However, a substantial number of respiratory acts were analyzed through semiautomatic software to facilitate the extrapolation process and minimize errors. Thirdly, COVID-19 patients were enrolled only in a few participating centers. Fourthly, the decision to transition from controlled to assisted breathing and to reassume controlled ventilation and/or increase sedation were not standardized between centers, reflecting institutional practices.

## 5. Conclusions

We identified patient characteristics (age, COVID-19) and treatment factors (P/F ratio_,_ days of sedation, dose of steroids before assisted ventilation) that are independently associated with excessive respiratory drive, effort, and transpulmonary driving pressure in patients with ARDS during the first transition to spontaneous assisted breathing. These factors also increase the need for sedation and the likelihood of resuming controlled ventilation. Our data suggest that careful evaluation of ICU treatment practices and patients’ respiratory drive and inspiratory effort is required to minimize the risk of self-induced lung injury and myotrauma.

## Figures and Tables

**Table 1:** Baseline characteristics and outcomes of patients’ study population.

Variables	n = 48
Age, yrs	58.2 (11.9)
Gender—male, n (%)	34 (70.8)
BMI, kg/m^2^	28.1 (5.6)
Underlying comorbidities	
Obesity, n (%)	14 (29.2)
Arterial hypertension, n (%)	25 (52.1)
Diabetes, n (%)	13 (27.1)
COPD, n (%)	9 (18.8)
Malignancy, n (%)	6 (12.5)
Chronic heart failure, n (%)	1 (2.1)
Chronic renal failure, n (%)	4 (8.3)
SOFA score	7.4 (3.3)
SAPS II score	37.5 (11.8)
Etiology	
COVID-19 pneumonia, n (%)	16 (33)
Outcomes	
Patients who went from PSV to ACV within 48 h, n (%)	3 (6.3)
Patients who went to RASS < −2 within 48 h, n (%)	9 (18.8)
Composite outcome †, n (%)	9 (18.8)
ICU mortality, n (%)	4 (8.3)
Mortality at 60 days after ICU admission, n (%)	3 (6.3)

† Composite outcome of patients who went from PSV to ACV or RASS lower than −2 within 48 h from the beginning of assisted spontaneous breathing. List of abbreviations: ICU: intensive care unit; BMI: Body Mass Index; COPD: Chronic Obstructive Pulmonary Disease; SAPS II: Simplified Acute Physiology Score II, SOFA: Sequential Organ Failure Assessment score.

**Table 2:** Ventilator settings, respiratory drive, and effort on the first day of spontaneous assisted breathing.

Variables	
Ventilator Setting	
Peak airway pressure (cmH_2_O)	17.5 [16.2–19.9]
PEEP (cmH_2_O)	8 [7.4–9.7]
VT/PBW (mL/kg)	7.4 [6–9.3]
Minute Ventilation (L/min)	9.9 [7.8–11.8]
Respiratory Rate (b/min)	20.5 [15.3–25.3]
Flow peak (L/s)	0.7 [0.7–0.9]
E_RS_ (cmH_2_O/mL)	19.9 [14.1–24.8]
R_RS_ (cmH_2_O/L/s)	12.4 [10.4–15.4]
ΔP_L,dyn_ (cmH_2_O)	16.2 [12.2–20.5]
Respiratory Drive	
P0.1 (cmH_2_O)	0.8 [0.4–1.9]
EAdi peak (µV)	9.6 [4.7–16.6]
Ti,_MECH_ (s)	1 [0.8–1.2]
Ti,_NEUR_ (s)	0.8 [0.6–1]
Inspiratory duty cycle (Ti/Ttot)	33.3 [27.7–38.4]
Neural inspiratory duty cycle (Tineu/Ttot)	0.3 [0.2–0.3]
PEIdyn (cmH_2_O/µV)	0.6 [0.4–1.8]
Neuroventilatory efficiency (mL/µV)	50.8 [29–99.9]
Respiratory Effort	
∆Pmus_EAdi_ (cmH_2_O)	−10.5 [−16.4–−5.1]
ΔPocc (cmH_2_O)	7.9 [3.8–11.1]
∆Pmus_ΔPocc_ (cmH_2_O)	7.9 [3.3–16]
PTP EAdi/min (cmH_2_O/s/min)	95.6 [38.9–175.3]
PTPEAdi/b (cmH_2_O/s)	4.9 [2.2–9]
Blood gas Exchange	
pH	7.4 [7.4–7.5]
PaCO_2_, mmHg	42 [39–48]
PaO_2_, mmHg	95 [86–116]
HCO3^−^, mEq/L	29 [27–32.8]

List of abbreviations: PEEP: positive end-expiratory pressure; VT/PBW: Tidal Volume per Predicted Body Weight; E_RS_: elastance of respiratory system; R_RS_: resistance of respiratory system; ΔP_L,dyn_: dynamic transpulmonary pressure P_0.1_: Airway Opening Pressure at 0.1 s, EAdi: electrical activity of diaphragm; ∆Pmus: delta muscle pressure; PEI: ∆Pmus/EAdi index; WOB: work of breathing; T_i,MECH_ = mechanical inspiratory time; T_i,NEUR_ = neural inspiratory time, PTP: Pressure–Time Product; PaCO_2_: partial pressure of carbon dioxide in arterial blood; PaO_2_: partial pressure of oxygen in arterial blood, in millimeters of mercury; HCO3^−^: bicarbonate concentration.

**Table 3:** Cox model of factors accounting for differences in composite outcome of transition from assisted to control ventilation or deep sedation (RASS ≤ −3).

Parameter	Unadjusted Hazard Ratio		Adjusted Hazard Ratio	
	*p*	HR (95% CI)	*p*	HR (95% CI)
COVID-19 (Ref = No-COVID)	0.0064	8.9 (1.85–43.2)	0.0502	6.96 (1–48.5)
Age	0.4785	0.98 (0.93–1.04)	-	-
Patient severity (SOFA)	0.0363	0.76 (0.6–0.98)	-	-
P/F ratio	0.0264	0.98 (0.97–1.00)	0.0884	0.99 (0.97–1.00)
Respiratory system compliance	0.1738	0.97 (0.93–1.01)	-	-
Days of deep sedation	<0.0001	1.12 (1.07–1.17)	0.0002	1.15 (1.07–1.24)
Days of NMBA	0.1104	1.04 (0.99–1.08)	-	-
Dose of steroids	0.0050	1.005 (1.002–1.009)	-	-
Days of mechanical ventilation	0.0762	1.02 (1.00–1.05)	-	-

## Data Availability

The datasets used and/or analyzed during the current study are available from the corresponding author upon reasonable request.

## Supplementary Materials

The following supporting information can be downloaded at: https://www.mdpi.com/article/10.3390/jcm13175227/s1, Figure S1: CONSORT flow diagram of study’s patients; Figure S2: Detailed information of patients enrolled at each centre; Figure S3: Distribution of P_0.1_ values for each breaths (Panel A). Two dashed lines identify three classes of respiratory drive: Low (P_0.1__vent_ < 1 cmH_2_O); Normal (P_0.1__vent_ 1–3.5 cmH_2_O) and High (P_0.1__vent_ > 3.5 cmH_2_O).: Distribution of EAdi_PEAK_ values for each breath (Panel B). Two dashed lines identify three classes of neuroventilatory drive d: Low (EAdi_PEAK_ < 5 µV); Normal (EAdi_PEAK_ 5–15 µV) and High (EAdi_PEAK_ > 15 µV); Figure S4: Distribution of respiratory effort by ∆Pmus_EAdi-derived_ values in the three cohorts of patients (Panel A). The dashed line identifies two classes of inspiratory effort: Normal (∆Pmus_EAdi-derived_ < 15 cmH_2_O) and High (∆Pmus_EAdi-derived_ > 15 cmH_2_O). Distribution of diaphragm efficiency by PTP/min values in the three cohorts of patients (Panel B). The dashed line identifies two classes of diaphragm efficiency: Low (PTP/min < 50 cmH_2_O/s/min); Normal (PTP/min 50–150 cmH_2_O/s/min) and High (PTP/min > 150 cmH_2_O/s/min); Figure S5: Flow chart of inclusion and exclusion criteria; Table S1: Ventilation setting at ICU admission and treatment factors before assisted ventilation; Table S2: Adjusted number of breaths by respiratory drive and effort classes and covid level estimate from GEE model; Table S3. Effects of patients’ baseline characteristics and treatment factors before transition to assisted ventilation on the proportion of breaths at higher class of respiratory drive (P0.1), inspiratory effort (∆Pmus_EAdi_) and lung distending pressure (ΔP_L,dyn_).

## References

[1] Yoshida T., Nakahashi S., Nakamura M.A.M., Koyama Y., Roldan R., Torsani V., De Santis R.R., Gomes S., Uchiyama A., Amato M.B.P. (2017). Volume-controlled Ventilation Does Not Prevent Injurious Inflation during Spontaneous Effort. Am. J. Respir. Crit. Care Med..

[2] Yoshida T., Torsani V., Gomes S., De Santis R.R., Beraldo M.A., Costa E.L., Tucci M.R., Zin W.A., Kavanagh B.P., Amato M.B. (2013). Spontaneous effort causes occult pendelluft during mechanical ventilation. Am. J. Respir. Crit. Care Med..

[3] Yoshida T., Amato M.B.P., Grieco D.L., Chen L., Lima C.A.S., Roldan R., Morais C.C.A., Gomes S., Costa E.L.V., Cardoso P.F.G. (2018). Esophageal Manometry and Regional Transpulmonary Pressure in Lung Injury. Am. J. Respir. Crit. Care Med..

[4] Grasselli G., Tonetti T., Protti A., Langer T., Girardis M., Bellani G., Laffey J., Carrafiello G., Carsana L., Rizzuto C. (2020). Pathophysiology of COVID-19-associated acute respiratory distress syndrome: A multicentre prospective observational study. Lancet Respir. Med..

[5] Ferrando C., Suarez-Sipmann F., Mellado-Artigas R., Hernandez M., Gea A., Arruti E., Aldecoa C., Martinez-Palli G., Martinez-Gonzalez M.A., Slutsky A.S. (2020). Clinical features, ventilatory management, and outcome of ARDS caused by COVID-19 are similar to other causes of ARDS. Intensive Care Med..

[6] Torregiani C., Baratella E., Segalotti A., Ruaro B., Salton F., Confalonieri P., Tavano S., Lapadula G., Bozzi C., Confalonieri M. (2024). Oscillometry Longitudinal Data on COVID-19 Acute Respiratory Syndrome Treated with Non-Invasive Respiratory Support. J. Clin. Med..

[7] Tonelli R., Fantini R., Tabbi L., Castaniere I., Pisani L., Pellegrino M.R., Della Casa G., D’Amico R., Girardis M., Nava S. (2020). Early Inspiratory Effort Assessment by Esophageal Manometry Predicts Noninvasive Ventilation Outcome in De Novo Respiratory Failure. A Pilot Study. Am. J. Respir. Crit. Care Med..

[8] Goligher E.C., Brochard L.J., Reid W.D., Fan E., Saarela O., Slutsky A.S., Kavanagh B.P., Rubenfeld G.D., Ferguson N.D. (2019). Diaphragmatic myotrauma: A mediator of prolonged ventilation and poor patient outcomes in acute respiratory failure. Lancet. Respir. Med..

[9] Goligher E.C., Dres M., Fan E., Rubenfeld G.D., Scales D.C., Herridge M.S., Vorona S., Sklar M.C., Rittayamai N., Lanys A. (2017). Mechanical Ventilation-induced Diaphragm Atrophy Strongly Impacts Clinical Outcomes. Am. J. Respir. Crit. Care Med..

[10] Spinelli E., Mauri T., Beitler J.R., Pesenti A., Brodie D. (2020). Respiratory drive in the acute respiratory distress syndrome: Pathophysiology, monitoring, and therapeutic interventions. Intensive Care Med..

[11] Brochard L., Slutsky A., Pesenti A. (2017). Mechanical Ventilation to Minimize Progression of Lung Injury in Acute Respiratory Failure. Am. J. Respir. Crit. Care Med..

[12] Goligher E.C., Dres M., Patel B.K., Sahetya S.K., Beitler J.R., Telias I., Yoshida T., Vaporidi K., Grieco D.L., Schepens T. (2020). Lung- and Diaphragm-Protective Ventilation. Am. J. Respir. Crit. Care Med..

[13] Vaporidi K., Akoumianaki E., Telias I., Goligher E.C., Brochard L., Georgopoulos D. (2020). Respiratory Drive in Critically Ill Patients. Pathophysiology and Clinical Implications. Am. J. Respir. Crit. Care Med..

[14] Alberti A., Gallo F., Fongaro A., Valenti S., Rossi A. (1995). P0.1 is a useful parameter in setting the level of pressure support ventilation. Intensive Care Med..

[15] Bellani G., Mauri T., Coppadoro A., Grasselli G., Patroniti N., Spadaro S., Sala V., Foti G., Pesenti A. (2013). Estimation of patient’s inspiratory effort from the electrical activity of the diaphragm. Crit. Care Med..

[16] Bertoni M., Telias I., Urner M., Long M., Del Sorbo L., Fan E., Sinderby C., Beck J., Liu L., Qiu H. (2019). A novel non-invasive method to detect excessively high respiratory effort and dynamic transpulmonary driving pressure during mechanical ventilation. Crit. Care.

[17] Goligher E.C., Fan E., Herridge M.S., Murray A., Vorona S., Brace D., Rittayamai N., Lanys A., Tomlinson G., Singh J.M. (2015). Evolution of Diaphragm Thickness during Mechanical Ventilation. Impact of Inspiratory Effort. Am. J. Respir. Crit. Care Med..

[18] Vaschetto R., Cammarota G., Colombo D., Longhini F., Grossi F., Giovanniello A., Della Corte F., Navalesi P. (2014). Effects of propofol on patient-ventilator synchrony and interaction during pressure support ventilation and neurally adjusted ventilatory assist. Crit. Care Med..

[19] Esnault P., Cardinale M., Hraiech S., Goutorbe P., Baumstrack K., Prud’homme E., Bordes J., Forel J.M., Meaudre E., Papazian L. (2020). High Respiratory Drive and Excessive Respiratory Efforts Predict Relapse of Respiratory Failure in Critically Ill Patients with COVID-19. Am. J. Respir. Crit. Care Med..

[20] Lassola S., Miori S., Sanna A., Menegoni I., De Rosa S., Bellani G., Umbrello M. (2023). Assessment of Inspiratory Effort in Spontaneously Breathing COVID-19 ARDS Patients Undergoing Helmet CPAP: A Comparison between Esophageal, Transdiaphragmatic and Central Venous Pressure Swing. Diagnostics.

[21] de Vries H.J., Jonkman A.H., de Grooth H.J., Duitman J.W., Girbes A.R.J., Ottenheijm C.A.C., Schultz M.J., van de Ven P.M., Zhang Y., de Man A.M.E. (2022). Lung- and Diaphragm-Protective Ventilation by Titrating Inspiratory Support to Diaphragm Effort: A Randomized Clinical Trial. Crit. Care Med..

[22] Pham T., Montanya J., Telias I., Piraino T., Magrans R., Coudroy R., Damiani L.F., Mellado Artigas R., Madorno M., Blanch L. (2021). Automated detection and quantification of reverse triggering effort under mechanical ventilation. Crit. Care.

[23] Di Mussi R., Spadaro S., Volta C.A., Bartolomeo N., Trerotoli P., Staffieri F., Pisani L., Iannuzziello R., Dalfino L., Murgolo F. (2020). Continuous assessment of neuro-ventilatory drive during 12 h of pressure support ventilation in critically ill patients. Crit. Care.

[24] Di Mussi R., Spadaro S., Mirabella L., Volta C.A., Serio G., Staffieri F., Dambrosio M., Cinnella G., Bruno F., Grasso S. (2016). Impact of prolonged assisted ventilation on diaphragmatic efficiency: NAVA versus PSV. Crit. Care.

[25] Telias I., Junhasavasdikul D., Rittayamai N., Piquilloud L., Chen L., Ferguson N.D., Goligher E.C., Brochard L. (2020). Airway Occlusion Pressure As an Estimate of Respiratory Drive and Inspiratory Effort during Assisted Ventilation. Am. J. Respir. Crit. Care Med..

[26] Telias I., Damiani F., Brochard L. (2018). The airway occlusion pressure (P(0.1)) to monitor respiratory drive during mechanical ventilation: Increasing awareness of a not-so-new problem. Intensive Care Med..

[27] Meinhardt J., Radke J., Dittmayer C., Franz J., Thomas C., Mothes R., Laue M., Schneider J., Brunink S., Greuel S. (2021). Olfactory transmucosal SARS-CoV-2 invasion as a port of central nervous system entry in individuals with COVID-19. Nat. Neurosci..

[28] Liu L., Liu H., Yang Y., Huang Y., Liu S., Beck J., Slutsky A.S., Sinderby C., Qiu H. (2012). Neuroventilatory efficiency and extubation readiness in critically ill patients. Crit. Care.

[29] Roze H., Repusseau B., Perrier V., Germain A., Seramondi R., Dewitte A., Fleureau C., Ouattara A. (2013). Neuro-ventilatory efficiency during weaning from mechanical ventilation using neurally adjusted ventilatory assist. Br. J. Anaesth..

[30] Boscolo A., Sella N., Pettenuzzo T., Pistollato E., Calabrese F., Gregori D., Cammarota G., Dres M., Rea F., Navalesi P. (2023). Diaphragm dysfunction predicts weaning outcome after bilateral lung transplant. Anesthesiology.

[31] Dres M., Schmidt M., Ferre A., Mayaux J., Similowski T., Demoule A. (2012). Diaphragm electromyographic activity as a predictor of weaning failure. Intensive Care Med..

[32] Meduri G.U., Bridges L., Shih M.C., Marik P.E., Siemieniuk R.A.C., Kocak M. (2016). Prolonged glucocorticoid treatment is associated with improved ARDS outcomes: Analysis of individual patients’ data from four randomized trials and trial-level meta-analysis of the updated literature. Intensive Care Med..

[33] Pham T., Heunks L., Bellani G., Madotto F., Aragao I., Beduneau G., Goligher E.C., Grasselli G., Laake J.H., Mancebo J. (2023). Weaning from mechanical ventilation in intensive care units across 50 countries (WEAN SAFE): A multicentre, prospective, observational cohort study. Lancet. Respir. Med..

[34] Kress J.P., Pohlman A.S., O’Connor M.F., Hall J.B. (2000). Daily interruption of sedative infusions in critically ill patients undergoing mechanical ventilation. N. Engl. J. Med..

[35] Sklar M.C., Madotto F., Jonkman A., Rauseo M., Soliman I., Damiani L.F., Telias I., Dubo S., Chen L., Rittayamai N. (2021). Duration of diaphragmatic inactivity after endotracheal intubation of critically ill patients. Crit. Care.

[36] Ely E.W., Truman B., Shintani A., Thomason J.W., Wheeler A.P., Gordon S., Francis J., Speroff T., Gautam S., Margolin R. (2003). Monitoring sedation status over time in ICU patients: Reliability and validity of the Richmond Agitation-Sedation Scale (RASS). JAMA.

[37] Bellani G., Laffey J.G., Pham T., Fan E., Brochard L., Esteban A., Gattinoni L., van Haren F., Larsson A., McAuley D.F. (2016). Epidemiology, Patterns of Care, and Mortality for Patients with Acute Respiratory Distress Syndrome in Intensive Care Units in 50 Countries. JAMA.

[38] Colebatch H.J., Greaves I.A., Ng C.K. (1979). Exponential analysis of elastic recoil and aging in healthy males and females. J. Appl. Physiol. Respir. Env. Exerc. Physiol..

[39] Akoumianaki E., Maggiore S.M., Valenza F., Bellani G., Jubran A., Loring S.H., Pelosi P., Talmor D., Grasso S., Chiumello D. (2014). The Application of Esophageal Pressure Measurement in Patients with Respiratory Failure. Am. J. Respir. Crit. Care Med..

