# Supplementary material for: Respiratory Drive, Effort, and Lung-Distending Pressure during Transitioning from Controlled to Spontaneous Assisted Ventilation in Patients with ARDS: A Multicenter Prospective Cohort Study
Source: J Clin Med. 2024 Sep 3;13(17):5227. doi: 10.3390/jcm13175227 (PMC11396025; doi:10.3390/jcm13175227)
Supplement: Supplementary file 1 [file jcm-13-05227-s001.zip › jcm-3144491-SI.pdf]

Respiratory drive, effort and lung-distending pressure during transitioning from controlled to spontaneous-assisted ventilation in patients with ARDS: a multicenter prospective cohort study.

Eleonora Balzani, Francesco Murgolo, Matteo Pozzi, Rossella Di Mussi, Nicola Bartolomeo, Umberto Simonetti,

Luca Brazzi, Savino Spadaro, Giacomo Bellani, Salvatore Grasso, Vito Fanelli

Electronic Supplementary Material

Corresponding Author:

Vito Fanelli MD, PhD
Associate Professor
Department of Surgical Sciences, University of Turin, Italy
Department of Anaesthesia, Critical Care and Emergency - Città della Salute e della Scienza Hospital – University of Turin, Italy
Corso Dogliotti 14, 10126 Torino, Italy.
Tel +39-011 633 4005 (office)
Fax +39-011 6960448
vito.fanelli@unito.it

Table S1. Ventilation setting at ICU admission and treatment factors before assisted ventilation

Variables	N=48
Minute ventilation, ml/min	8.8 (3.3)
VT/PBW, ml/kg	7.0 (1.8)
RR, bpm	19.3 (6.5)
PEEP, cmH_2O	12.1 (4.4)
P_{plat}, cmH_2O	24.4 (7.1)
PaO_2, mmHg	117.6 (42.6)
P/F ratio, mmHg	170.9 (89.0)
Driving pressure, cmH_2O	12.0 (5.5)
Respiratory system compliance, ml/cmH_2O	41.7 [27.5-57.5]
Rescue therapy before measurement, n (%)	26 (54.2)
Lung recruitment maneuvers, n (%)	10 (20.8)
Prone position, n (%)	22 (45.8)
Inhaled nitric oxide, n (%)	2 (4.2)
Days of deep sedation before measurement§, n	4.0 [2-8]
Days of NMBA before measurement, n	4 [1-6.5]
Dose of steroids‡ before measurement, mg	22.5 [7.5-60.0]
Patients receiving steroids, n (%)	38 (79.2)
Diagnosis of shock before measurement, n (%)	13 (27.7)
Days of mechanical ventilation before measurement, n	10.5 [7-16]

Data are expressed as mean (SD) or median [IQR]

List of abbreviations: VT: tida lvolume; PBW: predicted body weight; RR: respiratory rate; PEEP: positive end-expiration pressure; P_{plat}: plateau pressure; P/F ratio: ratio between arterial partial pressure of oxygen and fraction of inspired oxygen; PaO_2: partial pressure of oxygen in arterial blood; NMBA: neuromuscular blocking agent;

Table S2. Adjusted number of breaths by respiratory drive and effort classes and covid level estimate from GEE model

Parameter		Non-COVID	COVID	Effect of the parameter by covid level (p)
$EAdi_{PEAK}$ (μV)	<5	31.4±6.0	20.9±10.1	0.155
	5-15	42.2±4.9	35.3±9.2	
	>15	22.1±5.9	35.6±8.8	
$P0.1_{vent}$ (cmH_2O)	<1	58.9±5.3	29.6±7.6	<.001
	1-3.5	25.8±3.7	31.1±6.7	
	>3.5	10.3±4.5	20.5±7.5	
$\Delta Pmus_{EAdi\text{-}derived}$ (cmH_2O)	≤15	70.9±5.8	48.6±8.1	0.004
	>15	29.7±7.1	41.6±8.1	
ΔPL,dyn (cmH_2O)	≤15	65.9±7.6	36.9±9.9	0.043
	>15	67.4±7.7	79.8±10.7	

Data are shown as Least Square Means ± Standard Errors

Table S3. Effects of patients' baseline characteristics and treatment factors before transition to assisted ventilation on the proportion of breaths at higher class of respiratory drive (P0.1), inspiratory effort ($\Delta Pmus_{EAdi}$) and lung distending pressure ($\Delta P_{L,dyn}$).

**$P_{0.1}$ >3.5 cmH_2O**			
Variable		**Rate Ratio [95%CI]**	**p-value**
ARDS etiology	COVID vs. No-COVID	3.00 [2.43-3.71]	<0.0001
Days of NMBA	+1	1.01 [1.00-1.02]	0.1307
Dose of steroids‡	+10	1.013 [1.011-1.015]	<0.0001
**$\Delta Pmus_{EAdi}$ >15 cmH_2O**			
Variable			
ARDS etiology	COVID vs. No-COVID	1.84 [1.58-2.15]	<0.0001
Sex	M vs. F	0.65 [0.56-0.76]	<0.0001
Age	+10	0.83 [0.79-0.88]	<0.0001
Patient severity (SOFA)	+1	0.89 [0.87-0.91]	<0.0001
P/F ratio	+100	1.41 [1.32-1.51]	<0.0001
Respiratory system compliance	+10	0.69 [0.66-0.73]	<0.0001
Days of deep sedation§	+1	1.05 [1.03-1.06]	<0.0001
Dose of steroids‡	+10	1.02 [1.01-1.02]	<0.0001
Days of mechanical ventilation	+1	0.93 [0.92 -0.94]	<0.0001
**$\Delta P_{L,dyn}$ >15 cmH_2O**			
Variable			
ARDS etiology	COVID vs. No-COVID	1.48 [1.36-1.62]	<0.0001
Sex	M vs. F	0.80 [0.72-0.88]	<0.0001
Age	+10	1.10 [1.06-1.14]	<0.0001
P/F ratio	+100	0.92 [0.88-0.97]	0.0010
Respiratory system compliance	+10	0.97 [0.96-0.99]	0.0009
Dose of steroids‡	+10	1.002 [1.001-1.004]	0.0461

‡ We refer to steroids as the total cumulative equivalent dose of dexamethasone.
§ Days of sedation at Richmond Agitation Sedation Scale (RASS) >-3.

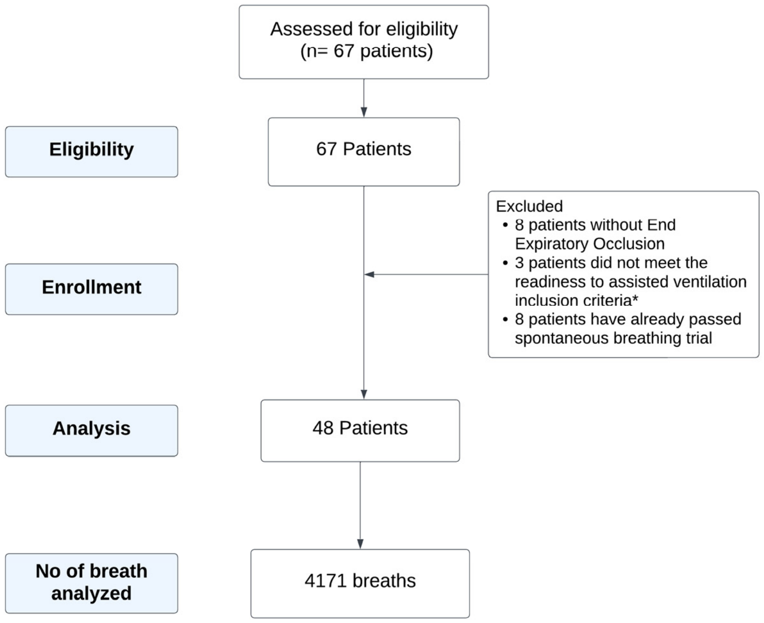

*Readiness to assisted ventilation is defined as a) improvement of the condition leading to acute respiratory failure; b) positive end-expiratory pressure (PEEP) lower than 10 cmH2O and inspiratory oxygen fraction (FiO2) lower than 0,5; c) Richmond agitation sedation scale (RASS) score between 0 and –2 and, d) ability to trigger the ventilator, i.e., to decrease pressure airway opening (PAO) >3–4 cmH2O during a brief (5–10 s) end-expiratory occlusion test.

Figure S1. CONSORT flow diagram of study's patients.

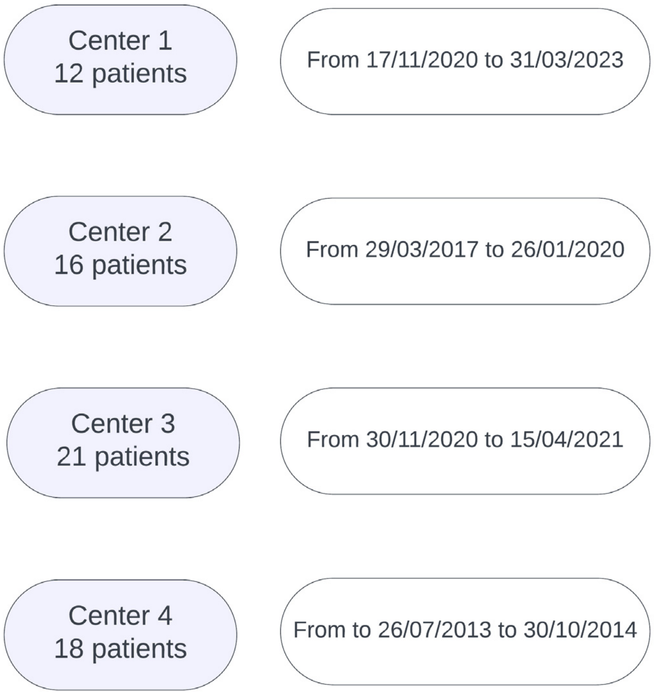

Figure S2. Detailed information of patients enrolled at each centre.

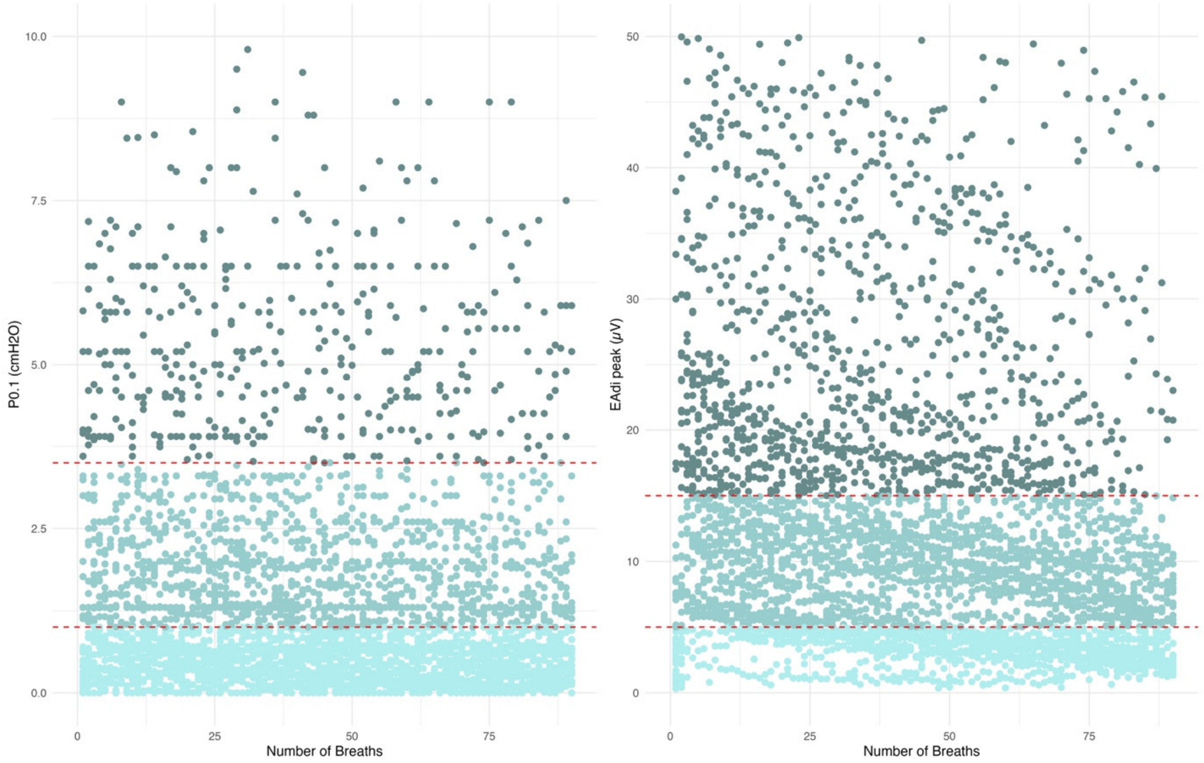

Figure S3. Distribution of $P_{0.1}$ values for each breaths (Panel A). Two dashed lines identify three classes of respiratory drive: Low ($P0.1_{vent}$ < 1 cmH_2O); Normal ($P0.1_{vent}$ 1-3.5 cmH_2O) and High ($P0.1_{vent}$ >3.5 cmH_2O).: Distribution of $EAdi_{PEAK}$ values for each breath (Panel B**)**. Two dashed lines identify three classes of neuroventilatory drive d: Low ($EAdi_{PEAK}$ <5 μV); Normal ($EAdi_{PEAK}$ 5-15 μV) and High ($EAdi_{PEAK}$ >15 μV).

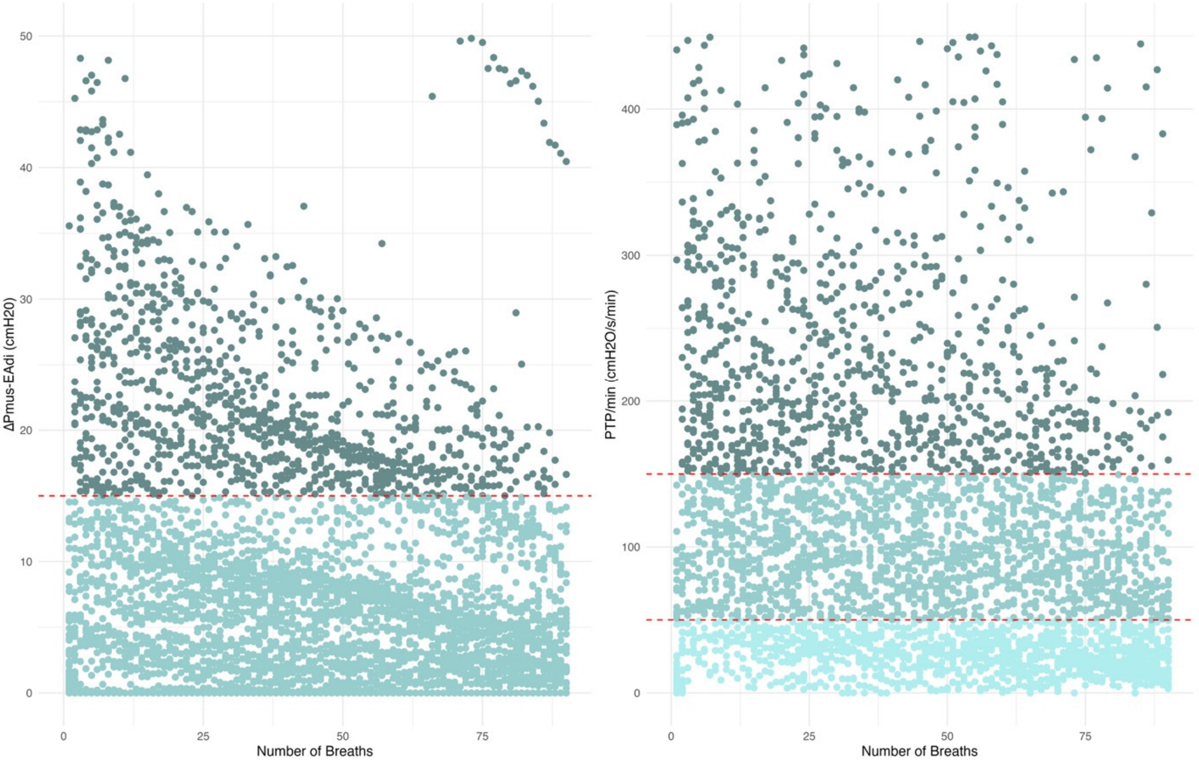

Figure S4. Distribution of respiratory effort by $\Delta Pmus_{EAdi\text{-}derived}$ values in the three cohorts of patients (Panel A). The dashed line identifies two classes of inspiratory effort: Normal ($\Delta Pmus_{EAdi\text{-}derived}$ < 15 cmH_2O) and High ($\Delta Pmus_{EAdi\text{-}derived}$ >15 cmH_2O). Distribution of diaphragm efficiency by PTP/min values in the three cohorts of patients (Panel B). The dashed line identifies two classes of diaphragm efficiency: Low (PTP/min < 50 cmH_2O/s/min); Normal (PTP/min 50-150 cmH_2O/s/min) and High (PTP/min >150 cmH_2O/s/min).

Inclusion Crtieria

- Diagnosis of ARDS
- Invasive mechanical ventilation for more than 72 hours
- Readiness to assisted ventilation:
  a) improvement of the condition leading to acute respiratory failure;
  b) PEEP lower than 10 cmH_2O and FiO_2 lower than 0.5;
  c) RASS) score between 0 and –2;
  d) ability to trigger the ventilator;
  e) hemodynamic stability.

Exclusion Criteria

- Neurological or neuromuscular pathology
- Phrenic nerve dysfunction
- Contraindication to the insertion of a nasogastric tube

Figure S5. Flow chart of inclusion and exclusion criteria.
